# A cluster randomised controlled trial of the clinical and cost-effectiveness of a 'whole systems' model of self-management support for the management of long- term conditions in primary care: trial protocol

**DOI:** 10.1186/1748-5908-7-7

**Published:** 2012-01-26

**Authors:** Peter Bower, Anne Kennedy, David Reeves, Anne Rogers, Tom Blakeman, Carolyn Chew-Graham, Robert Bowen, Martin Eden, Caroline Gardner, Mark Hann, Victoria Lee, Rebecca Morris, Joanne Protheroe, Gerry Richardson, Caroline Sanders, Angela Swallow, David Thompson

**Affiliations:** 1 Primary Care Research Group, Community Based Medicine, University of Manchester, 5th Floor Williamson Building, Oxford Road, Manchester M13 9PL, UK; 2 Institute of Primary Care and Health Sciences, Arthritis Research UK Primary Care Centre, Keele University, UK; 3 Centre for Health Economics, University of York, York YO10 5DD, UK; 4 Section GI Science, School of Translational Medicine- Hope, Clinical Sciences Building, Hope Hospital, Salford M6 8HD, UK

## Abstract

**Background:**

Patients with long-term conditions are increasingly the focus of quality improvement activities in health services to reduce the impact of these conditions on quality of life and to reduce the burden on care utilisation. There is significant interest in the potential for self-management support to improve health and reduce utilisation in these patient populations, but little consensus concerning the optimal model that would best provide such support. We describe the implementation and evaluation of self-management support through an evidence-based 'whole systems' model involving patient support, training for primary care teams, and service re-organisation, all integrated into routine delivery within primary care.

**Methods:**

The evaluation involves a large-scale, multi-site study of the implementation, effectiveness, and cost-effectiveness of this model of self-management support using a cluster randomised controlled trial in patients with three long-term conditions of diabetes, chronic obstructive pulmonary disease (COPD), and irritable bowel syndrome (IBS). The outcome measures include healthcare utilisation and quality of life. We describe the methods of the cluster randomised trial.

**Discussion:**

If the 'whole systems' model proves effective and cost-effective, it will provide decision-makers with a model for the delivery of self-management support for populations with long-term conditions that can be implemented widely to maximise 'reach' across the wider patient population.

**Trial registration number:**

ISRCTN: ISRCTN90940049

## Introduction

Long-term conditions are increasingly important determinants of quality of life and healthcare costs in populations worldwide [1]. There are major initiatives ongoing to improve quality of care and achieve better health in these populations [2]. Increasing healthcare costs associated with an ageing population and growing awareness of the links between behaviour and health has meant that even greater focus has been placed on self-management, defined as 'the care taken by individuals towards their own health and well being: it comprises the actions they take to lead a healthy lifestyle; to meet their social, emotional and psychological needs; to care for their long-term condition; and to prevent further illness or accidents' [3]. Self-management has the potential to improve population health without incurring major cost increases. In the UK, the influential Wanless report suggested that the future costs of healthcare were very much dependent on 'how well people become fully engaged with their own health' [4], although others have suggested self-management policies are part of an ideological shift from patient rights to individual responsibilities [5].

However, realising the potential of self-management requires effective ways of encouraging appropriate behaviour change in patients and professionals. There are a number of factors influencing self-management, including patient factors (*e.g*., lay epidemiology and health beliefs, self-efficacy, emotional responses to long-term conditions, identity, and pre-existing adaptations), and wider influences include the organisation of the healthcare system as well as access to material and community resources [6]. A number of models of self-management have been proposed in the literature, including increasing access to health information [7] and deployment of assistive technologies [8,9]. Patient skills training (through the Chronic Disease Self-Management Programme and its derivatives) is routinely used to encourage patients to access and use group-based support to enhance their individual self-management skills [10,11]. There is evidence for the effectiveness of the programme on some outcomes [12], but there are significant limitations. Intervention 'reach' is defined as the 'percentage and risk characteristics of persons who receive or are affected by a policy or program' [13,14]. Interventions with limited 'reach' are unable to translate the effectiveness of an intervention at the individual level to that of the wider population. In the case of the Chronic Disease Self-Management Programme, requirements for self-referral or referral from healthcare professionals means that levels of uptake can be low, and biased towards certain patient groups, threatening reach and equity [15,16].

### Enhancing the 'reach' of self-management

Health policy in the United Kingdom (UK) has worked with a model that organises care for long-term conditions around three tiers: self-management support for low risk patients; disease management for patients at some risk; and case management for patients with multiple, complex conditions. In the UK, the bulk of disease management is already delivered through primary care. Primary care is generally defined in terms of attributes such as a gatekeeping function and first contact care [17], but other attributes also make it an excellent platform for self-management support. Primary care offers open access between the health service and the population, can deliver continuity of care through an extended personal relationship or through informational continuity, and has a role in helping patients achieve care that balances compliance with clinical guidelines and consistency with patient needs and preferences. Delivering self-management support through primary care also maximises 'reach', because in any one year most patients with a long-term condition will consult their primary care provider.

However, there are major barriers to achieving effective self-management support in primary care. Self-management is only one priority among many facing primary care professionals [18], and there is evidence that many primary care professionals do not see self-management as a core part of their remit [19,20]. This is especially true when incentives (financial and otherwise) are focussed on specific clinical tasks and biomedical parameters [21]. Self-management has also been associated with new social movements and patient autonomy and activation and primary care may not necessarily or automatically harness this potential [22].

### Achieving the potential of primary care in delivering self-management support

Research in quality improvement in healthcare has indicated that, all other things being equal, multifaceted interventions are more effective than simpler ones [23], and that genuine and enduring change requires a multilevel approach, where interventions at different levels are interlinked and mutually reinforcing, in order to maximize impact. Our research team has engaged in a programme of research over a number of years that has explored the barriers and facilitators to effective self-management support. On the basis of this work, we argue that self-management support requires:

1. A whole systems perspective that involves interventions at the patient, practitioner, and service organisation levels in the delivery of self-management support. Many self-management interventions have focussed on patient behaviour change or professional training only, but we argue that each level has a different function in encouraging and supporting self-management behaviour, and that effects are maximised when interventions occur at all levels and include attention to patient actions outside the context of contacts with the health service [24].

2. Widening the evidence base to acknowledge a range of disciplinary perspectives on the way in which patients and professionals respond to and manage their long-term conditions. Although psychology has dominated the design of many interventions for self-management support through models such as self efficacy theory [10], there are a wide range of applied social science theories that can inform an understanding of the way that patients and professionals understand, respond to, and manage long-term conditions [25,26].

The Whole System Informing Self-Management Engagement (WISE) 'whole systems' model of self-management support has been designed to reflect these findings and provide a feasible and effective model of self-management support [27]. The model aims to support patients to receive guidance from trained practitioners working within a healthcare system geared up to be responsive to patient need. As a self-management research group in the National Primary Care Research and Development Centre, we developed the whole systems approach over a number of years as a response to the question, 'what can the NHS do to support self-management?' [28]. The whole systems approach had its roots in policy direction and analysis that was focussed on system elements, access, and patients, as well as professionals who inspired the direct transfer into self-management [29,30]. The current paper describes the model and a protocol for the evaluation of its implementation within a health system through a large-scale pragmatic cluster randomised controlled trial (RCT). Our approach follows the phased development and evaluation framework outlined for complex interventions by the Medical Research Council [31]. We have developed an evidence base for the elements of the WISE approach using mixed methodology; a combination of RCTs, nested qualitative studies and economic evaluation. In summary, the evidence shows that:

1. Information can be effectively improved to incorporate patient experience and expertise alongside medical information about management and treatment [32-36].

2. Clinician training in patient-centred consultation skills and shared decision-making with patients to guide and support self-management is acceptable and appropriate and leads to positive outcomes [37-40].

3. Health services systems that are better aligned to patient practices of self-management are generally well received [41,42].

**Figure 1:**
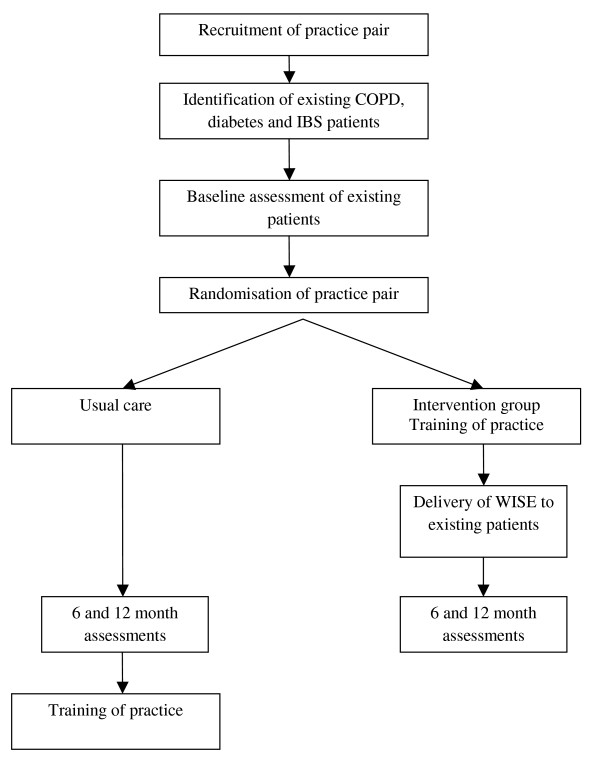
**Study flowchart (original design-see discussion)**.

The WISE intervention as described in this protocol is a culmination of the findings and learning from this body of work; we have made modifications according to the evidence, *e.g*., our findings on the limits to patient centredness [43], and we have adapted the clinical training element so that it can be delivered to a primary care team, and this has been previously evaluated in an exploratory study [44]. The study is a pragmatic cluster RCT using a wait list control (Figure 1). The intervention is designed to impact on all primary care staff in a practice, thus randomisation is at the level of the practice to avoid contamination. However, the aim of the study is to improve outcomes at the level of the individual patient.

## Methods

The trial is a pragmatic, two-arm, practice-level cluster RCT evaluating outcomes and costs associated with the WISE 'whole systems' model.

The principal research question is: Does the adoption of the WISE 'whole systems' model of self-management support in primary care lead to improved health outcomes and cost-effective management of patients with long-term conditions, compared to routine primary care?

### Population

The general practice is the cluster in the trial, and we aim to involve all practices in a local 'health economy' in the UK. The context is a primary care organisation (a Primary Care Trust) in Salford, in the North West of England. The organisation has a strong commitment to supporting self-management, viewing this as part of a strategic approach to improving the health and well-being of the population. Many factors related to living in Salford may lead to poor health:

'Life expectancy for people who live in Salford is less than in the rest of England and Wales. Salford's men die on average three years earlier and women die just less than three years earlier, compared with the national average. The reasons for this are varied and include social factors, such as high deprivation rates, high unemployment and a reliance on social housing in particular areas.' [45]

We aim to recruit patients with three long-term conditions: diabetes; chronic obstructive pulmonary disease (COPD) and irritable bowel syndrome (IBS). The WISE 'whole systems' model is designed to be robust and adaptable enough to use with the vast majority of patients with a long-term condition. The particular conditions to be included in the trial have been chosen on the basis of a number of theoretical, policy, and practical criteria, and they have important similarities and differences. Each condition is amenable to self-management interventions [40,46,47], and there is already a significant evidence base that will facilitate comparison of the effects of the WISE 'whole systems' model with alternatives (such as individual patient education and the Chronic Disease Self-Management Programme). The conditions are also of sufficiently high prevalence within practices to meet the sample size needs of the proposed trial. UK health policy has identified the need to reduce health inequalities [48], and local data suggest that the prevalence of COPD increases with deprivation.

The conditions also have important differences in symptoms, experience, and management. IBS is a more 'contested' condition with greater disagreement about diagnosis and appropriate management. The management of both diabetes and COPD are also incentivised in the UK under the Quality and Outcomes Framework [49], whereas IBS is not.

As a pragmatic trial of a model of service delivery, the inclusion criteria are simple and broad to enhance the external validity of the study. Patients must have a clinical diagnosis of COPD, diabetes, or IBS, identified from existing primary care systems using appropriate clinical registers (for COPD and diabetes) and Read codes (for IBS) and verified by the primary care professionals. Patients must also demonstrate sufficient English to be able to complete questionnaires; and there must be agreement from the practice that the patient is appropriate for research assessment. Exclusion criteria include patients in the palliative care stage of condition or the presence of mental health problems that reduce capacity to consent and participate.

Patients who have two or more of our index conditions, or a single index condition and another long-term condition are still included in the trial. Where a patient is identified as having two or more of the three trial conditions, clinical staff in the practice will be asked to determine which the main condition is, in order to assist with appropriate outcome measurement (see below).

### Interventions

**Figure 2:**
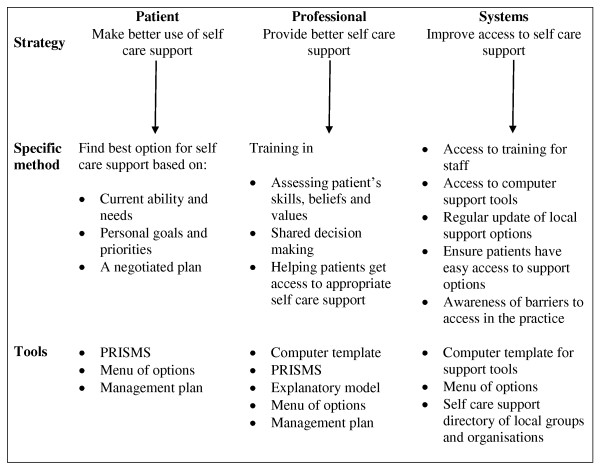
**Outline of the WISE whole systems model**.

**Figure 3:**
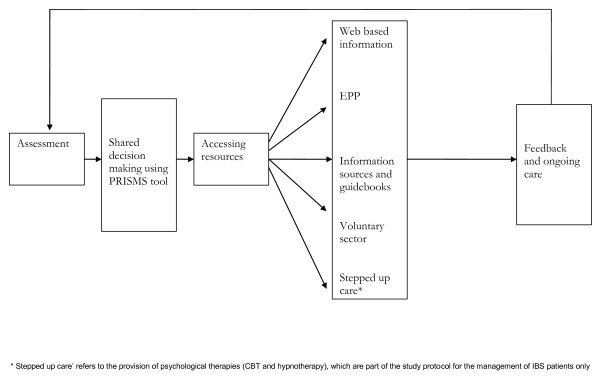
**Process of care in the WISE model**.

The intervention is designed to impact on the patient, professional, and system levels (see Figure 2). The primary target of the intervention is the cluster (practice), and the overall aim of the intervention is to encourage practices to adopt a structured and patient-centred approach to the routine management of long-term conditions through providing skills, resources, and motivation to make changes to service delivery in line with the principles of the WISE 'whole systems' model (see Figure 3). The development and evaluation of the training intervention have taken place prior to this trial, and details of the theoretical background and content have been published elsewhere [44]. The planned approach to training combines evidence-based approaches to changing professional behaviour with approaches to 'normalise' those behaviours in current practice. The intervention involves the whole practice, and there are also 'system' links to the local health organisation (the Primary Care Trust), which provides access to additional resources, including a dedicated website of local self-management support.

**Figure 4:**
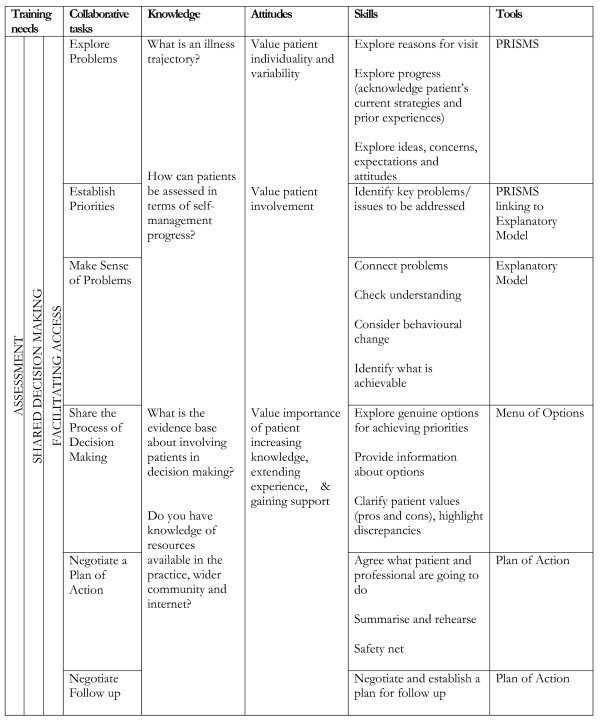
**Collaborative tasks, knowledge, attitudes, skills and support tools in WISE**.

Practice-based training seeks to teach three core skills to primary care staff (see Figure 4):

1. Assessment of the individual patient's self-management support needs, in terms of their current capabilities and current illness trajectory.

2. Shared decision making about the appropriate type of self-management support based on that assessment (*e.g*., support from primary care, written information sources, long-term condition support groups or condition-specific education), facilitated by the PRISMS tool [50].

3. Facilitating patient access to support. This may involve signposting patients to various resources depending upon the outcomes of the assessment and shared decision-making processes. These may include access to the Expert Patients Programme (the UK version of the Chronic Disease Self-Management Programme), disease specific courses (such as pulmonary rehabilitation classes), or generic support such as befriending. The training encompasses ways health professionals can negotiate with patients about more appropriate utilisation of health service resources. In the case of IBS, this may also involve referral to psychological treatment services (cognitive behavioural therapy and hypnotherapy) for eligible patients (so-called 'stepped-up care'). Patients with IBS are informed of the possibility of referral to such services through information leaflets.

**Figure 5:**
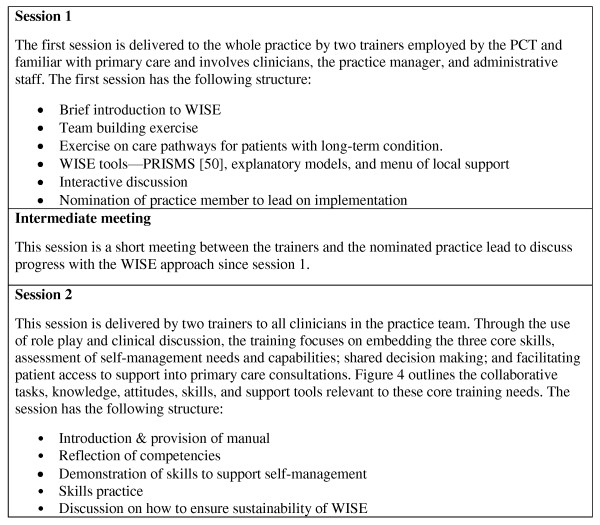
**Training sessions**.

As part of the training, primary care professionals receive specific assistance in development of the core WISE skills, followed by integration of techniques through role-play, and individualised performance feedback based on that role-play [51,52]. The intervention is delivered over two sessions with an intermediary session to discuss progress and involves all relevant staff within the practices for the first session (including general practitioners (GPs), nurses, practice managers, and reception staff) and clinical staff for the second session (Figure 5). A training manual is given to all those who participate in the training for use within the training session and to support practice. The training was piloted and modified on the basis of a pilot [44]. The intervention is conducted by trained facilitators working alongside the research team rather than the research team itself, to test a model of delivery that would be feasible in routine practice and wider implementation.

### Outcomes

All outcomes are at the level of the individual patient. Each practice sends eligible patients a questionnaire at baseline, to be returned directly to the research team. Follow-up questionnaires are sent at 6 and 12 months. The primary endpoint will be the 12-month follow-up of patient health outcomes and costs. The trial has three primary outcomes based on patient-reported measures: shared decision-making with GPs; self-efficacy; and generic health-related quality of life (Additional file 1). These represent core measures along the 'causal pathway' from intervention to health outcomes. The study will also collect a number of secondary outcomes, including disease-specific quality of life, self-management behaviours, service utilisation, empowerment, general health, social/role limitations, well-being, and vitality (Additional file 1). There is no blinding of patients or outcome assessors, although all outcomes are self-report. The analyst remains blind to allocation.

### Design

The study is a pragmatic cluster RCT using a wait list control (Figure 1). The intervention is designed to impact on all primary care staff in a practice, thus randomisation is at the level of the practice to avoid contamination.

### Practice recruitment and randomisation

Practices are recruited via practice visits and asked to identify their preferred time during the year for training. Practices are paired as closely as possible according to their preferred times, and using a minimisation procedure, one practice in each pair allocated to training in the first year, with the other practice allocated to training at the same time the following year. Research staff recruiting practices are unaware of the next allocation in the sequence at the time of recruitment. Baseline (and subsequent follow-up) data collection then takes place at both practices in a pair at the same time. This ensures a balance between the intervention and control groups, thus avoiding potential bias from changes to care delivery outside the trial context (*e.g*., new government policy or local system changes on the management of long-term conditions).

The practice (cluster) pairs are allocated--one to the intervention group and the other to the control--using a minimisation algorithm by the trial statistician. Minimisation variables are practice size, practice deprivation (measured by the Index of Multiple Deprivation), and practice contractual arrangements (*i.e*., general medical services or personal medical services). This ensures that, barring attrition, the two arms of the trial will have equal numbers of practices and be balanced on the minimisation variables and data collection timeline.

After practice pairing and allocation, potentially eligible patients at each intervention and control group practice are identified from the computer systems and checked for eligibility to be contacted by practice clinical staff.

### Sample size

Sample size calculations have been made on the basis of data collected from the national evaluation of the Expert Patients Programme [11]. Although all three patients groups will be combined in the primary analysis (see below), we have powered the trial to detect a fairly small effect of the intervention on diabetes, COPD, and IBS separately.

Data on outcomes from the national evaluation of the Expert Patients Programme had a range of intraclass correlation coefficients (ICCs) from 0.01 to 0.07. For the power calculations, we assume an ICC of 0.05. Baseline-follow up correlations are taken to be 0.6, towards the lower end of those found in the Expert Patients Programme. On these assumptions, each arm of the trial requires 18 practices, and 36 patients per condition per practice, to achieve 80% power to detect an effect size of 0.21 per condition. To allow for attrition of practices (estimated to be around 10%), we aim to recruit 20 practices into each arm of the trial. Questionnaires will be sent to 80 patients per practice with each condition. For each of the three conditions, this should provide on average 48 patients per practice at baseline, reducing to 36 patients at 12 months. Smaller practices may not have 80 patients with COPD, in which case we will compensate by recruiting additional patients from larger practices. On the basis of the above, we aim to recruit totals of 1,728 diabetic, 1,728 COPD, and 1,728 IBS patients.

**Figure 6:**
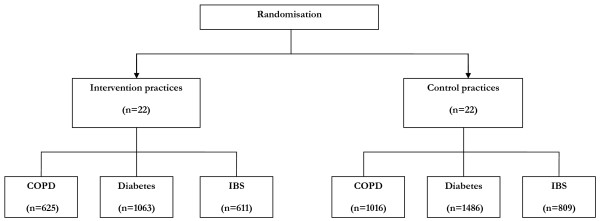
**CONSORT diagrams (baseline data only)**.

The trial is still ongoing, but we present our baseline sample in Figure 6.

### Training procedure

Intervention practices will receive training as soon as possible after baseline data collection and subsequent trial arm allocation; control practices will receive training one year later. Training will be undertaken in two sessions, with the second session approximately one month after the first.

### Analysis

An *a priori *analysis plan--laying down full details of the primary and secondary outcomes, covariates, treatment of missing values, other methods, and sensitivity analyses--will be drawn up prior to analysis of the RCT.

Each outcome will be subjected to analysis of covariance within a multilevel multivariate regression framework, following intention-to-treat principles and with the analyst blind to practice allocation to trial arms. A two-level mixed model will be used to account for the clustering of patients within practices and for variation between practice means. The primary analysis will test for an intervention effect across all three condition groups combined, adjusted for any main effects of condition on outcome and controlled for baseline values of the outcome and the design factors (practice list size, Index of Multiple Deprivation (IMD), and type of contract). Although practices are randomised in pairs, the pairing is purely to balance the groups with respect to the data collection timeline, and practice pairs can vary in all other respects. Consequently, the pairing will not be a covariate in the analysis.

A secondary analysis adding in an interaction term between treatment group and condition will be conducted to determine if the intervention effect differs by condition. If the interaction is statistically significant, condition-specific effect sizes and associated 95% confidence intervals will be derived. Additional secondary analyses will be undertaken to examine the impact of the intervention on quality of life within each of the three target condition groups, as measured by each of the condition-specific instruments (Additional file 1).

Sensitivity analyses will be undertaken to assess the stability of the results to the model specification. In particular, to patient and practice covariates found to be not fully balanced across the two trial arms, to the treatment of missing values, and to normality assumptions where outcomes demonstrate non-normal distributions.

The trial will include an economic analysis to compare the costs and outcomes for the trial arms. Cost-effectiveness analysis will be used to estimate the incremental costs and quality adjusted life years (QALYs). Appropriate sensitivity analysis will be used to assess the robustness of results to changes in key parameters. Discounting will be conducted as appropriate. This analysis will be to determine whether WISE provides a cost-effective use of resources when compared with current alternatives. This will be a 'within trial' cost-effectiveness analysis where the costs and effects of the WISE intervention over the time period of the trial will be compared with usual care comparators described above.

We will use a modification of the service utilisation questionnaire successfully developed by us for our national evaluation of the Expert Patients Programme [53] that measures primary healthcare (GP visits, practise nurse visits), community health and social care, secondary healthcare services, out-of-pocket costs, and costs of lost productivity. Unit cost estimates will then be applied to these data to generate patient-level cost estimates. The sources of unit costs will include routine or published literature such as Personal Social Services Research Unit Costs of Health and Social Care [54]. Patient-level costs will be appropriately discounted. A GP cost per minute will be derived, and a cost per consultation for each group will be estimated.

For the economic analysis, our measure of effectiveness is the QALY, a composite measure of health combining the quantity and quality of life [55]. QALYs will be generated using the EQ-5D instrument that will be collected via patient questionnaire at each follow up point [56]. QALYs will be estimated for each patient by weighting their survival time using the EQ-5D weights [56]. The mean difference in costs and effect between the treatment options will provide an informative incremental cost effectiveness ratio. However, economic evaluation is conducted under conditions of uncertainty. Parameter imprecision will be included in the analysis using probabilistic sensitivity analysis based on Monte Carlo simulation [57]. The results of the simulations will illustrate the uncertainty in the data and will be represented using cost effectiveness acceptability curves (CEACs) [58,59]. Given the data from the trial, the CEAC will plot the probability of the WISE intervention being more cost effective than usual care for a range of threshold values of an additional QALY.

### Wider process evaluation

A process evaluation has been designed to complement and provide additional information concerning the trial. Details about the process evaluation and accompanying qualitative study are included in Additional files 2 and 3.

## Discussion

The proposed study is an ambitious project that is designed to provide a pragmatic, yet rigorous assessment of the benefits of a whole systems approach to the delivery of self-management support in primary care.

The size and complexity of the proposed evaluation means that it is extremely challenging to implement, and initial delivery of the intervention and research components has faced a number of barriers that has led to a number of minor changes to the protocol. We detail these changes and their potential threats to internal and external validity below.

### Single health economy

Our aim was to deliver the proposed intervention across all practices in a single primary care organisation to assess an intervention effect across a complete health economy. Although we were able to recruit 32 practices in Salford (73% response rate), this did not give us the desired level of statistical power. We therefore spread our recruitment to a neighbouring Primary Care Trust (Bury). This area has a similar socio-economic profile, and the overall spread of IMD scores across practices is quite similar to Salford (4.5 to 65.5, compared to 6.6 to 77.2). In addition, the two Trusts shared the same chief executive and both are part of a North West self-management initiative. However, some aspects of the whole systems intervention detailed previously were not available (such as existence of a dedicated self-management education team and availability of certain community-based support schemes). Differences in the financial arrangements between Trusts also meant that we were unable to offer control group practices in Bury PCT the intervention after 12 months.

### Selection of patients before practice allocation

One of the threats to the validity of a cluster randomised trial is recruitment bias, where professionals allocated to different trial arms recruit differently depending on their allocation, leading to selection bias and baseline incomparability [60]. It is preferable in these cases to recruit patients prior to allocation. Although this was our intention, in the event we found that practices required adequate advance notice of their training date, hence it became necessary to inform them of their group allocation prior to patient selection. This does raise the possibility of bias, but we are confident that such bias will be small. Initial patient selection was via existing disease registers and Read codes; therefore, the only way practices could influence recruitment was to request exclusion of a patient after they had been identified through these methods. These exclusions represented a relatively small proportion of patients (11% control and 15% intervention with COPD, 10% and 11% respectively for diabetes, and 18% and 11% respectively for IBS).

### Baseline sampling and assessment

Response rates from patients at the practices first to enter the trial were considerably lower than originally anticipated. In view of this, we made a number of adjustments to improve response. To ensure the patient numbers required for our target level of statistical power, we increased the number of patients surveyed to include all eligible patients at each practice, up to a maximum of 200 per condition (selected at random where this applied). Very few practices had more than 200 patients for any condition, hence the study became, in effect, a total population survey.

We also introduced a financial incentive to patients for returning a completed questionnaire. There is some evidence that questionnaire length is a determinant of response rates [61], and we took the decision to change the baseline questionnaire to focus on a smaller number of core variables (see Additional file 1). The main effect on the trial of the shortened baseline questionnaire is to reduce our scope for analysis of baseline moderators of treatment effect. However, such analyses are always secondary to the primary intention-to-treat analysis.

## Competing interests

The authors declare that they have no competing interests.

## Authors' contributions

PB wrote the first draft of the paper and all other authors read and approved the final manuscript. DR developed the trial analysis and GR developed the economics analysis. AK, CCG, TB, and JP developed the training interventions. AR, AK, RB, VL, CS, and RM developed the process evaluation and qualitative study.

## Supplementary Material

Additional file 1 **Outcome measures**. Details of the outcome measures used in the trial [62-85]. Click here for file

Additional file 2 **Process Evaluation**. Outline of the process evaluation [86,87]. Click here for file

Additional file 3 **A qualitative study of the role of patients' social networks for long-term condition management in primary care**. Outline of the qualitative study [88-90]. Click here for file
