# Supplementary material for: A cluster randomised controlled trial of the clinical and cost-effectiveness of a 'whole systems' model of self-management support for the management of long- term conditions in primary care: trial protocol
Source: Implement Sci. 2012 Jan 26;7:7. doi: 10.1186/1748-5908-7-7 (PMC3274470; doi:10.1186/1748-5908-7-7)
Supplement: Additional file 1 — Outcome measures. Details of the outcome measures used in the trial [62-85]. [file 1748-5908-7-7-S1.DOC]

**Outcome measures**

**Primary Outcomes**

*Shared decision making (baseline, 6 and 12 months)*

The WISE approach is designed to assist patients and professionals to come to a shared understanding of the optimal ways of managing long-term conditions that takes into account the patient’s preferred management strategies and priorities regarding outcomes. Shared decision making will be measured using the 6-item short-form Health Care Climate Questionnaire, which measures autonomy support provided by professionals (*e.g.*, provision of choice, acknowledging the individual’s perspective) [62-65].

*Self-efficacy (baseline, 6 and 12 months)*

Self-efficacy refers to a self perception of confidence to complete activities. It will be measured using a 5-item scale of self efficacy to undertake chronic disease management developed for this purpose [66] and used in previous trials of the Chronic Disease Self-management programme and the Expert Patients Programme.

*Health related quality of life (baseline, 6 and 12 months)*

The EQ5D is a generic measure of health related quality of life that allows the generation of QALYs. The 5-item scale covers mobility, self care, usual activities, pain, anxiety and depression, each with three levels of severity and provides a utility value based on a population tariff, where zero equates to dead and one equates to full health [56].

**Secondary outcomes**

*Health status*

We include a selection of sub-scales from the Medical Outcomes Survey: general health; social/role limitations; energy and vitality; and well-being. These scales were used in our previous analysis of the EPP [11].

*Empowerment (6 and 12 months)*

Empowerment may be defined as ‘an enabling process or outcome arising from communication with the health care professional and a mutual sharing of resources over information relating to illness, which enhances the patient’s feelings of control, self-efficacy, coping abilities and ability to achieve change over their condition’. Interventions to improve shared decision making should theoretically improve patient empowerment. Empowerment will be measured using the Patient Enablement Questionnaire, a 6-item scale which examines patient perceptions that their consultations with health professionals enable them to understand their problem, cope, and keep themselves healthy [67,68].

*Health behaviour (6 and 12 months)*

A key theoretical question in self-management research is whether self-management interventions achieve their effects through changes in health behaviour, or through other mechanisms such as changes in self efficacy [69]. To explore this issue, self-management behaviour will be measured using a modified scale of 11 generic types of self-care activity [70], supplemented by a small number of disease-specific additional activities [71-73].

*Management options*

Guidance and subsequent use of appropriate self care support options are a key part of the WISE approach. This study will use a modified version of a question from the Patient Assessment of Chronic Illness Care (PACIC) questionnaire [74] to which we have added options relating to specific local programmes and encouragement received from primary care professionals to use guidebooks, web-sites, support groups or health trainers.

*Condition specific quality of life (6 and 12 months)*

Disease specific quality of life in COPD will be measured using the Short Breathing Problems Questionnaire, a shortened 10-item version of the Breathing Problems Questionnaire which measures the effects of COPD on mobility, self care, social activities energy and mental health [75,76].

Disease specific quality of life in diabetes will be measured with the Diabetes Quality of Life questionnaire (DQOL) [77], a 15-item scale measuring issues such as satisfaction with treatment, impact on function and relationships, and self care.

Disease specific quality of life in IBS will be measured using the IBS-QOL, a 34-item scale with eight subscale scores, including dysphoria, interference with activity, body image, health worry, food avoidance, social reaction, sex, and relationships [78]. We will be using a shortened version with 16 items.

*Service utilisation (baseline, 6 and 12 months)*

We will use a modification of the service utilisation questionnaire from the national evaluation of the Expert Patients Programme [11] which measures primary health care (GP visits, pharmacy), community health and social care, secondary health care services, out of pocket costs and costs of lost productivity. Unit cost estimates will be applied to these resource use data to generate patient level cost estimates.

**Additional measures**

Some additional items will be included in the questionnaire not as outcomes but rather as possible moderators of the effect of the intervention.

*Illness perceptions (6 and 12 months)*

Illness representations are implicit mental models of illness, which are related to coping strategies and outcomes [79]. One of the best known measures of illness representations is the Illness Perceptions Questionnaire. This study uses the brief 8-item IPQ, excluding the causal scale [80,81].

*Health literacy*

Health literacy is the ‘degree to which individuals have the capacity to obtain, process and understand basic health information and services needed to make appropriate health decisions’ [82] and may place a limit on the ability of patients to engage with self-management. We will use questions on the patient’s ability to read and understand health literature.

*Social capital (6 and 12 months)* Research indicates that social capital may have an influence on care pathways and management of illness [83]. All three domains of social capital (social networks, neighbourhood attachment & civic participation) are significantly associated with poor measures of health status [84]. Social capital will be measured with 14 items from the Health Survey for England (2003).[85]
