# Supplementary material for: A cluster randomised controlled trial of the clinical and cost-effectiveness of a 'whole systems' model of self-management support for the management of long- term conditions in primary care: trial protocol
Source: Implement Sci. 2012 Jan 26;7:7. doi: 10.1186/1748-5908-7-7 (PMC3274470; doi:10.1186/1748-5908-7-7)
Supplement: Additional file 2 — Process Evaluation. Outline of the process evaluation [86,87]. [file 1748-5908-7-7-S2.DOC]

**Process Evaluation**

*Background*

The approach used for development and evaluation of WISE follows the phased framework for complex interventions outlined by the Medical Research Council. The pre-clinical and development phase was informed by theory and research evidence and set in the context of policy expectations and guidance. The next phase was to evaluate the feasibility of the training intervention in the clinical setting and a formative evaluation approach was used to refine training prior to roll-out in a randomized controlled trial [44]. A process evaluation has been designed which will complement and provide additional evidence to the main trial.

The theoretical and conceptual framework for development and evaluation of the WISE approach has been twofold. Firstly, the WISE approach has been developed from evidence derived from mixed methods research to investigate the circumstances and components within which patient-centred self-management is likely to be most effective. Secondly, Normalisation Process Theory (NPT) [86,87] has been developed from existing evaluation studies and as a conceptual framework has utility in sensitizing the research to the reaction, incorporation or rejection of WISE from a service user and professional perspective. The success (or failure) of interventions is predicated on the potential for embedding new interventions within normal ‘everyday’ practices.

*Process evaluation*

*What are the barriers and facilitators which affect the implementation of WISE at patient, clinical and organisational levels?*

The process evaluation will investigate how far the knowledge of the intervention has been diffused, taken up, adapted locally and embedded at the level of Practice-based Commissioning (PBC) consortia and the Primary Care Trust, and what the consequences of any such diffusion may have been. It will determine how broadly acceptable the WISE approach is to recipients.

Where results are positive, evidence is needed to identify ‘active ingredients’, to aid generalisability and to facilitate learning and translation into everyday practice. Similarly, where results are negative or inconclusive, evidence is needed to identify sources of failure and stasis, especially cultural, organisational and behavioural reasons why promising theory has not translated into practice. It is important to identify what works well for which practices, individuals and stakeholders and in what context.

Aims

1. To explore patient perspectives about and engagement with existing service management arrangements and the nature of interaction with professionals and service engagement.
2. To explore patient attitudes to engagement with new self-management arrangements.
3. To examine changes in personal management arrangements including impact on existing caring relationships and use of additional services and resources.
4. To explore professionals’ and patients’ attitudes and responses to the perceptions of the costs and benefits to patients and professionals of implementing WISE.

Methods

A multi-method approach will be carried out in parallel to the main trial.

- Observations of consultations between clinicians and patients and of the practice context
- Face to face in-depth interviews with key stakeholders: patients, the WISE trainers, trained practitioners (GPs and nurses), practice staff, CBT therapists and hypnotherapists
- Baseline and follow-up interviews with a purposive sample of relevant members of PBC consortium and PCT governance bodies
- Collation of documents generated by the training
- Field-notes from researchers attending practices and training notes (written by the trainers after each training sessions)
- Post-training evaluation questionnaire
- Questionnaire survey of training participants based on NPT constructs to be conducted 6-months post-training

*Sampling*

Observations of consultations with patients with relevant long-term conditions were carried out pre and post-training during the feasibility study. Up to 30 patients will be purposefully sampled for interview based on baseline data. All practices taking part in the training within Salford PCT will be invited to take part in interviews and interviews will continue until a broad representation of practice types is reached. Practice staff to be interviewed will include all clinicians, the practice manager and a member of the administrative staff. Both trainers and both therapists will be interviewed.
