# Supplementary material for: A cluster randomised controlled trial of the clinical and cost-effectiveness of a 'whole systems' model of self-management support for the management of long- term conditions in primary care: trial protocol
Source: Implement Sci. 2012 Jan 26;7:7. doi: 10.1186/1748-5908-7-7 (PMC3274470; doi:10.1186/1748-5908-7-7)
Supplement: Additional file 3 — A qualitative study of the role of patients' social networks for long-term condition management in primary care. Outline of the qualitative study [88-90]. [file 1748-5908-7-7-S3.DOC]

**A qualitative study of the role of patients’ social networks for long-term condition management in primary care**

This project was a qualitative study embedded within the exploratory trial. It is a related but separate project exploring how the self-management support provided through WISE via clinicians and health services interacts with the wider support from family, friends and the community, with an eventual aim of ensuring that they mutually engage an individual with self- management. Much health related social network research has focused on the impact of social networks in the genesis of long-term health conditions and associated inequalities; yet there has been little research on the role of social networks in their ongoing management. The management of chronic illness is complex, involving the interplay of a variety of personal and contextual factors. The relationship between health behaviours, resources and support within a patient’s everyday life needs to be considered within the context of contact with clinicians and health services [88]. It is through social networks that health problems are acknowledged, formal health care is sought and ongoing management occurs [83] These informal and formal networks of support and resources overlap to influence long term condition management. It is necessary to understand how patients’ normalise new skills and interactions into their everyday lives within the context of health services and the influence this has on help seeking and engagement with self-management activities such as diet, medication use or physical activity.

*Aims*

- To identify what constitutes a social network for people with long term conditions
- To explore the interaction of lay and professional networks in shaping the meaning of self-management
- To investigate how changes in social networks and access to resources over a one year period influences health management and utilisation of primary care

*Methods*

A 1-year qualitative longitudinal study has been conducted with initial face-to-face interviews, bi-monthly telephone follow-up interviews. In a final face-to-face interview an adapted version of a network mapping technique [89] was used to identify the social network structure which was then explored within the interview. The quality of the networks and the resources available from network members were then explored through an in-depth interview.

*Sampling*

Participants were recruited from the exploratory phase of the trial. Participants have been purposefully sampled [90] to have at least one of three conditions: and had either diabetes (n=15), COPD (n=7) or IBS (n=8). We also sampled to include a range of ages and length of time since diagnosis.
